# Stem Cell-Derived Models of Viral Infections in the Gastrointestinal Tract

**DOI:** 10.3390/v10030124

**Published:** 2018-03-10

**Authors:** Wyatt E. Lanik, Madison A. Mara, Belgacem Mihi, Carolyn B. Coyne, Misty Good

**Affiliations:** 1 Department of Pediatrics, Washington University School of Medicine, St. Louis, MO 63110, USA; wlanik@wustl.edu (W.E.L.); maram@wustl.edu (M.A.M.); belgacem.mihi@wustl.edu (B.M.); 2 Department of Pediatrics, University of Pittsburgh School of Medicine, Pittsburgh, PA 15224, USA; coynec2@pitt.edu; 3 Center for Microbial Pathogenesis, Children’s Hospital of Pittsburgh of UPMC, Pittsburgh, PA 15224, USA

**Keywords:** enteroids, organoids, intestinal stem cells, mini-guts, enteroviruses

## Abstract

Studies on the intestinal epithelial response to viral infection have previously been limited by the absence of in vitro human intestinal models that recapitulate the multicellular complexity of the gastrointestinal tract. Recent technological advances have led to the development of “mini-intestine” models, which mimic the diverse cellular nature and physiological activity of the small intestine. Utilizing adult or embryonic intestinal tissue, enteroid and organoid systems, respectively, represent an opportunity to effectively model cellular differentiation, proliferation, and interactions that are specific to the specialized environment of the intestine. Enteroid and organoid systems represent a significant advantage over traditional in vitro methods because they model the structure and function of the small intestine while also maintaining the genetic identity of the host. These more physiologic models also allow for novel approaches to investigate the interaction of enteric viruses with the gastrointestinal tract, making them ideal to study the complexities of host-pathogen interactions in this unique cellular environment. This review aims to provide a summary on the use of human enteroid and organoid systems as models to study virus pathogenesis.

## 1. Introduction

The intestinal epithelium is a complex system, with both structural and cellular characteristics that allow a unique balance between nutrient absorption and crucial host defense. In order to facilitate adequate fluid and nutrient uptake, the intestinal epithelium forms a lattice-like structure made of villi and crypts for increased surface area. The single layer of intestinal epithelial cells retains the absorptive and secretory capabilities while maintaining a rapid cell turnover in order to provide a physical barrier between the luminal microenvironment and the host. Intestinal stem cells are an integral driving force in facilitating gut barrier integrity by producing the terminally differentiated epithelial cell types that compose the intestinal epithelia, including enterocytes, goblet cells, Paneth cells, and enteroendocrine cells [1,2,3]. Each of these cell types plays a critical role in host immunity and defense against pathogens [4,5].

Enteric viruses remain one of the leading causes of acute gastroenteritis among both developing and developed countries [6]. However, despite their significant impacts on human morbidity and mortality, there is limited knowledge surrounding many aspects of gastrointestinal (GI) physiology and viral pathogenesis that occur during viral infections of the GI tract. Conventional in vitro models have been unsuccessful in investigating some aspects of viral infections of the intestine due to the inability of standard cell culture systems to recapitulate the complex environment within the GI tract. With the development of intestinal enteroid and organoid systems, recent studies have demonstrated a novel method that models the unique environment of the intestine. This advancement in research methodology opens new possibilities to study a variety of biological aspects of enteric viruses. 

Despite the risk that enteric viruses pose, standard culturing methods have prevented a complete assessment of many mechanisms utilized by these viruses to infect the intestinal barrier. For example, difficulties associated with viral propagation in transformed cell lines and/or animal models hindered norovirus research for many years. Here, we summarize advances in the development of intestinal enteroids and organoids as well as the differences between these model systems. We also describe recent literature using these mini-intestine models to study enteric viruses, mainly rotaviruses, norovirus, adenoviruses and enteroviruses. Although more research is required before effective therapies may be established for worldwide use, human enteroids and organoids have provided an exciting new advance to study enteric viral infections in vitro.

## 2. Defining Gastrointestinal Models

In recent years, the development of mini-intestinal cultures has provided functional in vitro models with the ability to robustly proliferate and recapitulate basic intestinal morphology. Due to slight variations between different mini-intestine systems, namely—enteroids and organoids—nomenclature distinguishing between them has been described by the National Institutes of Health (NIH) Intestinal Stem Cell Consortium (ISCC). Organoids are defined as a three-dimensional hollow enterosphere that form a single layer of epithelium with apical-basal polarity that originates from induced pluripotent stem cells (iPSCs) or embryonic stem cells [7,8,9]. Another factor specific to organoids is the underlying mesenchymal cell layer that develops in organoid culture, which is critical for the maintenance of epithelial tissue identity [7,8,10,11,12,13]. The development of organoids follows a pattern similar to embryonic intestinal development, allowing them to be useful in developmental studies [8,10,11,12,14,15]. In contrast, enteroids originate from small intestinal crypt stem cells and specifically lack mesenchymal cells, such as myofibroblasts, fibroblasts, and stromal cells. Despite lacking mesenchyme cell types, they retain the ability to proliferate [1,16]. Enteroids represent mature epithelia, allowing the modeling of homeostasis and the investigation of various disease mechanisms [11,17,18,19]. Both enteroids and organoids, represent genetically variable models between individuals, which allows for disease and susceptibility studies as well as genetic manipulations to further study disease states [16,18,20,21].

## 3. Development of Mini-Intestinal Models

### 3.1. Enteroid Development

Within the last decade, advances in the isolation and maintenance of intestinal stem cells has allowed the generation and long-term culture of mini-intestines in the laboratory. Sato et al. [1] and Ootani et al. [22] first described methods to isolate and culture intestinal epithelial stem cells, thus developing a novel crypt culture system. Hans Clevers and colleagues developed specific conditions for culturing mouse intestinal crypts including the minimum growth factors required to support enteroids, specifically R-spondin, epidermal growth factor (EGF) and Noggin [1]. In enteroid culture systems, the Wnt pathway is the key signaling pathway that promotes stem cell proliferation and maintains undifferentiated stem cells [23,24]. R-spondin is one of the components that is a necessary Wnt signaling enhancer [25]. Additionally, EGF plays an integral role in enteroid culture due to its ability to stimulate intestinal stem cell proliferation [26]. Noggin is another media component essential for continued passaging of crypt cultures [1]. Noggin has been shown to inhibit bone morphogenetic protein (BMP) signaling, which restricts the proliferative capacity of stem cells [27,28]. Finally, laminin-rich Matrigel is used as a basement membrane to support the intestinal crypt growth, since laminin (α1 and α2) are found at high concentrations at the bottom of murine intestinal crypts [29]. In combination, the above growth factors and laminin-rich Matrigel can support a long-term culture system of murine intestinal epithelial crypts [1]. With the establishment of the murine intestinal enteroid systems, studies have been conducted to examine potential limitations of this model. For instance, Fuller et al. investigated factors such as the age of mice and region of murine intestine [30]. Their study demonstrated that all murine age groups and tissue sections were able to produce enteroids. Additionally, they determined that mouse intestinal enteroids retain proliferative capacity over time following cryopreservation, indicating their capability for long-term storage [30].

A key difference between murine enteroids and human enteroids is the requirement of exogenous Wnt as a growth factor. Since Wnt is endogenously produced at high concentrations by mouse Paneth cells, exogenous Wnt is not necessarily needed to maintain mouse enteroids; however, this is not the case for human enteroids, which require Wnt to culture human crypts successfully [31]. Because Wnt is naturally produced by mouse enteroids, they are less challenging and readily expand in vitro [32]. 

More recently, an L-cell line was derived to secrete the primary growth factors Wnt3a, R-spondin 3, and Noggin (L-WRN) as a highly concentrated conditioned medium that has been used to rapidly expand human intestinal stem cell populations as well as to maintain an undifferentiated progenitor phenotype in human enteroid cultures [33]. By using an equal mixture of L-WRN conditioned medium and primary cell culture media, human enteroids exhibit an increase in cell number in ileal enteroids [33]. The combination of critical growth factors in conditioned media enables enteroid cultures to proliferate quickly and efficiently. Once cryopreservation was established as a functional method of storage for human enteroids, Saxena et al. created a human intestinal enteroid bank obtained from various regions of the intestine including the duodenum, jejunum, and ileum [21]. Overall, the ability to collect and store intestinal enteroids long-term facilitates further investigation and allows for modeling of many human intestinal diseases in vitro.

### 3.2. Organoid Development

The culture of intestinal organoids is temporally associated with growth factor requirements based on gastrulation and definitive endoderm formation to promote a posterior development [7,8,34,35]. A high concentration of activin A for 3 to 4 days, is required to induce Sox17 and Foxa2, transcription factors that coordinate the development of the endoderm lineage as reviewed in [36]. As in enteroid models, activation of the Wnt pathway is of critical importance to promote the development of mouse embryonic stem cells into a posterior intestinal state. In human organoid models, however, Cao et al. showed that fibroblast-conditioned medium was required to induce a posterior fate as shown by the high expression of the colon marker CDX2 [35], through the activation of fibroblast growth factor (FGF) and Wnt pathways [7,8]. Interestingly, these studies demonstrated that prolonged exposure to FGF and Wnt for 4 days is necessary to produce an irreversible CDX2 expression and intestinal specification of human organoids [7]. In line with these findings, it was shown that to promote a posterior human intestinal organoid development, human iPSCs were exposed to activin A for 3 days, then cultured for 4 days with Wnt3a and FGF4 to induce posterior intestinal specificity, and allowed to grow for 14 to 28 days into intestinal organoids [7,8]. Human iPSCs in this stepwise process of differentiation mimic the process of embryological development as they become intestinal organoids [7,8,36].

## 4. Mini-Intestinal Differentiation and Morphology

### 4.1. Region-Specific Differentiation

The GI tract is divided into multiple segments, each with unique functional properties. For instance, the duodenum is the only small intestinal segment to express duodenal cytochrome b reductase (CYBRD1), which is involved in iron metabolism [37]; the jejunum is the main expresser of lactase phlorizin hydrolase (LCT), which is a disaccharidase that facilitates absorption of lactose [38,39]; and the ileum solely produces the sodium-bile acid transporter (SLC10A2) to facilitate bile acid absorption [40]. In studies aiming to investigate if region-specific genes are conserved in enteroids, Middendorp et al. performed RNA sequencing on villi and crypts from murine intestinal segments and corresponding murine enteroids [41]. Importantly, the gene expression of mouse enteroids was similar to those of the in vivo intestinal segments for *CYBRD1*, *LCT*, and *SLC10A2*, respectively [41]. Many of the genes were conserved: duodenum 78% and 43%, jejunum 70% and 65%, and ileum 65% and 49%, respectively [41]. VanDussen et al. measured gene expression between duodenum, ileum, and enteroids, as well as rectal tissue and colonoids from humans [33]. In addition, they found that sucrase-isomaltase (*SI*) and *SLC10A2* were specifically induced in ileal enteroids, which corresponded to ileal tissue expression [33]. Transient receptor potential cation channel, subfamily V, member 6 (*TRPV6*) a calcium transport protein, was preferentially expressed in the duodenum and in duodenal enteroids compared to other intestinal sections and their corresponding enteroids [33,42]. The gene expression of human rectal tissue and colonoids did not correlate with the gene expression found in small intestinal tissues nor enteroids. Specific rectal markers such as carbonic anhydrase 1 (*CA1*) were only induced in the rectal-derived colonoids and corresponded to in vivo rectal expression [33]. These studies show that both murine and human enteroids retain a region-specific identity between in vivo intestinal sections and in vitro enteroid culture [33,41]. 

Region-specific expression can also be induced in organoid cultures as shown by the directed differentiation of human iPSCs into intestinal organoids and chronological manipulations of integral growth factors to simulate the embryonic intestinal development [7]. By using FGF4 and Wnt3a as growth factors, Spence et al. promoted the formation of colonic organoids identified by the transcription factors KLF5 and SOX9, two intestinal markers, and CDX2 [7]. In addition, Wang et al. determined that duodenal, jejunal, and ileal stem cells grown using air-liquid interface culture from human fetal intestine express genes based on anatomical location [43]. For instance, cultured duodenal stem cells expressed the genetic markers *TFF2* and *Muc5AC*, which are typical gastric epithelial markers, while cultured jejunal stem cells expressed intestinal markers, such as *Muc2*. In the same way, cultured ileal stem cells resulted in the formation of an epithelium similar to the colon [43]. These studies demonstrate that organoid development follows region-specific differentiation depending on tissue source [43] or can be induced to recreate specific regional identity upon growth factor manipulation [7,43].

### 4.2. Crypt-Villus Development

**Figure 1:**
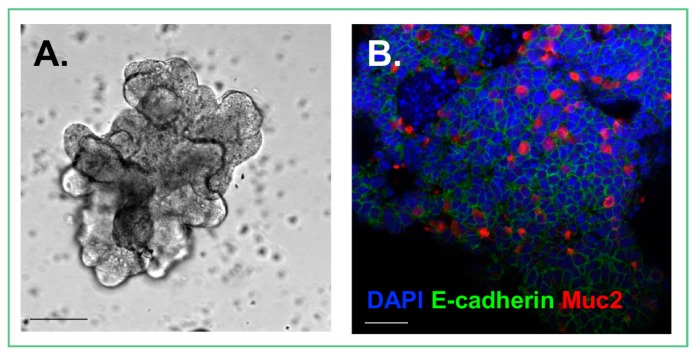
Cultured enteroid. (**A**) Photomicrograph demonstrating the architecture of an enteroid; (**B**) Confocal image of an enteroid stained for DAPI (Blue), E-cadherin, an epithelial cell marker (Green), and Muc2, a goblet cell marker (Red). Size bars = 50 μm.

In the small intestine, villi function to increase surface area and contain cell types that contribute to the unique environment of the small intestine. The absorptive and secretory cells of villi arise from surrounding intestinal crypts (Figure 1), where the proliferative cells of the intestinal epithelium are located. During in vitro growth of small intestinal enteroids, crypt budding occurs and results in the development of the crypt-villus axis over time [1]. The crypt-villus axes generated from a developing enteroid is reminiscent of a mature intestine in an in vitro enteroid culture [1]. Several recent studies have microengineered scaffolds for the cultivation of enteroids in an attempt to mimic the structure of the intestine [44,45,46]. One such study has shown that seeding crypts on an artificial polylactic-glycolic acid (PLGA)-rich scaffold, can foster the generation of epithelial structures resembling villi both in vitro and after implantation into small and large animals [44]. Wang et al. demonstrated that a cross-linked collagen hydrogel supported the proliferation and differentiation of primary human enteroids after being molded into villi-shaped columns [45]. Additionally, human intestinal enteroids cultured in the presence of primary intestinal myofibroblasts using a silk-based cylindrical scaffold, were able to generate a microvilli brush border as well as tight junctions after differentiation [46]. By using artificially engineered scaffolds, these experiments have shown an ability to produce apical brush borders, tight junctions, and epithelial cell differentiation into relevant cell types required to recreate the physiological role of the intestine.

Organoid culture systems provide an alternative method to evaluate the development of crypt-villus axes. This was shown when human fetal intestinal stem cells produced epithelial folds after 10 days of air-liquid interface culturing, with duodenum and jejunum producing a finer pattern of folding than the ileum [43]. By histological examination of the intestinal stem cell culture, villi-like structures are present with proliferative cells located proximal to the basement membrane with more pronounced structure forming from the ileum [43]. Thus, air-liquid interface culturing of fetal stem cells could ultimately maintain the morphological and cellular features of the fetal intestine. 

Implanting human intestinal organoids in the kidney capsule of a mouse produced distinct crypts and villi resembling mature small intestine, suggesting that vasculature along with the host environment can affect organoid growth [13]. Based off of this previous work, Finkbeiner et al. implanted approximately 3-week-old human intestinal organoids into immunocompromised mice to elucidate if organoids can mature into adult-like tissue [10]. The implanted organoids formed an architecture which was similar to adult intestine with villi and a mesenchymal layer, unlike the control organoids that lacked a crypt-villus architecture [10]. These studies proposed that organoids offer a surrogate method to induce villus formation and that organoids require the mesenchyme and vasculature to properly organize epithelial cells into villi. 

A fundamental factor in villus formation relates to peristalsis-like motion, which is difficult to recreate in the laboratory setting. Donald Ingber’s group recently demonstrated that this can be accomplished in a gut-on-a-chip microfluidic device that has the ability to be subjected to peristalsis-like mechanical stress [47]. Human primary intestinal epithelium was grown in a microfluidic device subjected to a constant flow of media and a vacuum pump. This provides both shear and mechanical stress, thus, enabling small intestinal villi-like structures to develop [48]. Strikingly, epithelial cells grown in culture without the consistent flow of media did not develop into 3-dimensional villi-like formations, but instead maintain a monolayer of differentiated cells [48]. Intestinal enteroid fragments cultured in the presence of intestinal microvascular endothelial cells were more confluent than cultures of single intestinal stem cells, enteroid fragments, and single intestinal stem cells plus the microvascular endothelial cells [48]. Kasendra et al. showed that the gut-on-a-chip using human enteroid fragments and intestinal microvascular endothelial cells promote intestinal villi-like structures, cell lineage differentiation, epithelial barrier integrity, apical brush borders and resemble the adult duodenal transcriptome [48]. These experiments indicate that fluid flow and mechanical stress can influence cellular and physical characteristics of progenitor cells in vitro, and the inclusion of mesenchymal cells can model the microenvironment in the intestine. 

### 4.3. Cell Types within Crypts

The most common cells that reside at the bottom of intestinal crypts are crypt base columnar (CBC) cells, interspersed by Paneth cells [49]. In order to identify the specific CBC cells as the progenitor for the epithelial cell types found in the intestinal epithelium, Hans Clevers and colleagues used leucine-rich repeat-containing G-protein-coupled receptor 5 green fluorescent protein (Lgr5-GFP) reporter mice to trace the cell lineage of Lgr5^+^ intestinal stem cells by harvesting murine crypts and plating individual cells [1]. In cells expressing the *Lgr5* gene, enteroids formed more efficiently, while Lgr5^−^ cells did not form enteroids [1]. After two weeks, enteroids from single cells were indistinguishable from enteroids derived from whole crypts. Confocal and electron microscopy demonstrated that Paneth cells and stem cells occupied the bottom of the crypts, while goblet cells and enteroendocrine cells lined the lumen of the enteroids [1]. Notably, there were no mesenchymal cell types observed. Taken together, these studies demonstrate that Lgr5^+^ intestinal stem cells are the progenitor cell for epithelial cell differentiation [1]. 

As Lgr5^+^ intestinal stem cells differentiate, they move upward to occupy the +4 position of the intestinal crypt, above the Paneth cells, and become transit amplifying (TA) cells that are rapid cycling precursors to the other terminally differentiated epithelial cell types as reviewed in [50,51]. Recent evidence has shown that when Lgr5^+^ stem cells are damaged, specific TA cells such as enterocytes and secretory progenitors, can trigger crypt regeneration by reverting to a multipotent cell state [3,52,53]. For example, Bmi1^+^ quiescent endocrine cells, with the help of Paneth cell precursors, are able to convert to an Lgr5^+^ intestinal stem cell identity when there is damage to the native Lgr5^+^ stem cell population [54]. In addition, TA cells at higher positions in the crypt, position 6 and above, have been found to restore the loss of Lgr5^+^ stem cells due to radiation [55]. 

Of note, Paneth cells are important to the stem cell niche environment, where they produce critical growth factors for the proliferation and maintenance of Lgr5^+^ intestinal stem cells [56]. Paneth cells also secrete a myriad of antimicrobial peptides and proteins, which are crucial for host defense and the innate immune system [5]. Paneth cells are unique when compared to other intestinal stem cell lineages as they migrate down into the crypt, instead of up and out of the crypt as the other terminally differentiated epithelial cells. Rare TA cells that reside in the +4 position and maintain a long S phase called label-retaining cells are recognized as precursors of terminal Paneth cells [3]. The diversity of cell types and cellular environments within crypts is one of the hallmark features of enteroid and organoid modeling that enables a wide variety of experimentation.

## 5. In Vitro Models to Study Viral Infections

### 5.1. Human Intestinal Organoids to Model Rotaviral Infections

Rotavirus (RV) is a segmented, double-stranded RNA virus that is one of eight species in the *Reoviridae* family [57]. The most common species, *Rotavirus A,* is the foremost cause of severe gastroenteritis in children between the ages 2 to 5 worldwide, with 25 to 70 percent leading to hospitalization [6,58]. Thus, the development of in vitro models that can recapitulate the human intestine is crucial for expanding current knowledge on RV pathogenesis. Both intestinal enteroids and organoids have been shown recently to serve as in vitro models to investigate both animal RV (ARV) and human RV (HRV) infection of the GI tract. 

Conventional in vitro methods for culturing RV include propagating the virus into stable cell lines, such as Rhesus kidney (MA104), human colorectal adenocarcinoma (HT-29 and Caco-2), or into primary cells, which require multiple passages to reach sufficient growth for analysis [59,60,61,62,63]. With the development of induced pluripotent stem cells into mini-intestines or human intestinal organoids, the ability to observe both ARV and HRV infection in a novel model has been demonstrated [21]. Finkbeiner et al. first used human intestinal organoids as in [7,8] to cultivate ARV using Rhesus rotavirus (RRV; strain G3P[3]) and observed efficient replication by immunofluorescence microscopy for viral replication factories [64]. This study also found that cleavage of the RRV structural protein 4 (VP4) by pancreatin, a key digestive enzyme, facilitated RRV infection in epithelial cells, but also in mesenchymal cells, which had not been previously recognized as a target for RV infection [64]. 

After establishing that human intestinal organoids support ARV infection, Finkbeiner et al. explored whether this model could also support HRV replication by using HRV-infected stool [64]. Stool samples collected from Texas Children’s Hospital between 2002 and 2010 were used to inoculate human intestinal organoids, with active replication of HRV confirmed by assessing the presence of viral RNA (vRNA) and viroplasms [64]. Importantly, the levels of vRNA generated in human intestinal organoids were approximately 10 times greater than isolates grown in MA104 cells, indicating that human intestinal organoids supported better viral growth as compared to immortalized cell lines [64]. Taken together, these data indicate that human intestinal organoids are a more suitable model to study rotavirus. 

### 5.2. Human Intestinal Enteroids to Model Rotaviral Infection

In testing human intestinal enteroids as a model to culture HRV, Saxena et al. infected human intestinal enteroids derived from adult duodenal, jejunal, and ileal biopsies with multiple HRV strains including Ito (G3P[8]) and Wa (G1P[8]) and compared these strains to RRV strain G3P[3] [21]. Strikingly, based on multiple parallel readouts for viral replication (including flow cytometry, qRT-PCR, electron microscopy, and immunohistochemistry), the authors observed high levels of HRV infection, with less robust infection by RRV [21]. Immunofluorescence-based analyses indicated that HRV primarily infected E-cadherin-positive enterocytes, with infection also identified in chromogranin A-positive enteroendocrine cells, suggesting that HRVs exhibit a cell-type specificity of infection [21]. 

Other assays have also been performed in human intestinal enteroids to investigate possible physiological effects during HRV infections, such as cytotoxicity assays, swelling assays, and enteroid genotyping. Common in HRV infection, luminal swelling and fluid movement occur during the diarrheal process. To test if human intestinal enteroids can mimic this process, they were exposed to the HRV strain Ito and the cross-sectional area was measured via microscopy over several hours [21]. As a result, the lumen of infected human intestinal enteroids expanded between 3 to 4 h post inoculation (hpi) and reached maximum radius by 6 hpi [21]. Similarly, when exposed to rotaviral enterotoxin NSP4, which acts via chloride secretion, significant luminal expansion occurred [21]. These results indicated that human intestinal enteroids are functional and physiologically active in the presence of HRV infection. 

### 5.3. Cultivating Norovirus in Human Intestinal Enteroids

Human Norovirus (NoV, previously known as “Norwalk-like virus”) is a non-enveloped, icosahedral, single-stranded RNA virus that remains a significant source of acute gastroenteritis and is responsible for sporadic community or common-source outbreaks [6,65]. Despite its impact on human health, little is known regarding NoV infections of the human GI tract due to the inability to propagate NoVs in previously existing human-based models. Although enteric bacteria were shown to enhance human NoV infection of B cell lines (M12, WEHI-23, BJAB) [66], it remained unclear whether human NoVs also replicated in enterocytes or other intestinal-associated cell types. After many attempts by others to infect cell lines with NoV, the development of human intestinal enteroids has provided an exciting new model to further study human NoV development, signaling, and pathogenesis. In a study by Ettayebi et al., the most common genotype of human NoV, GII.4, was derived from fecal filtrates and inoculated in human intestinal enteroids [67,68]. By 96 hpi, there was a 1.5–2.5 log_10_ increase in viral progeny, as indicated by the presence of vRNA. Cytopathic effects, such as cell rounding, destruction of the monolayer, and an increase in nonviable cells were also observed in GII.4-infected cultures [68]. Further evidence of viral replication was confirmed by immunofluorescence, flow cytometry, and confocal microscopy. Detection of the enterocyte marker villin in NoV VP1-positive cells confirmed that enterocytes are the likely primary cell target for human NoV replication [68]. 

Human NoV is a genetically diverse pathogen classified into three genotypes: GI, GII, and GIV. Within these genotypes, they are further divided into 9 GI genotypes and 20 GII genotypes [68]. Studies have indicated that genetically determined factors, such as secretor status, can influence susceptibility to certain genotypes of human NoV. Secretor-positive status is indicated by having the active form of the fucosyltransferase 2 enzyme, which mediates the transfer of fucose onto histoblood group antigen (HBGA) precursors in GI cells [69]. Ettayebi et al. demonstrated that a GII.4 human NoV strain was able to infect human enteroids obtained from secretor-positive individuals, yet unable to infect those from secretor-negative individuals [68]. Furthermore, a GII.3 strain of human NoV was capable of infecting some, but not all, secretor-negative human enteroids. These data indicate host restriction to specific genotypes of human NoV. 

Environmental factors have also been shown to play a role in NoV infection. In one study, three strains of human NoVs (GII.3, GII.17, and GI.1) were pretreated with nontoxic doses of human bile, which is abundant in the small intestine and involved in digestion and absorption [68]. Interestingly, the authors found that bile was required for NoV infection of human enteroids, whereas untreated enteroids were not capable of supporting infection [68]. Taken together, these studies support that human intestinal enteroids, possibly in conjunction with bile, provide a suitable in vitro model of human NoV infections. 

### 5.4. Enteroids as a Model of Enterovirus Infection

Enteroviruses are a common source of human GI infections transmitted via the fecal-oral route, yet little is known of their infection pathogenesis within the intestine [62]. In experiments conducted by Drummond et al., three enteroviruses (coxsackievirus B (CVB), echovirus 11 (E11), and enterovirus 71 (EV71)) successfully infected human premature intestinal enteroids and resulted in activation of antiviral signaling pathways [62], previously unobserved in conventional cell line-based culturing systems. Importantly, this study found that whereas the enteroviruses tested did infect human enteroids, there were virus type-specific differences in the induction of host antiviral and pro-inflammatory signaling in response to infection, with E11 inducing the greatest levels of host defense. Concomitantly, E11 also induced the greatest cytotoxic effect, accompanied by the disruption of tight junctions, as well as increased levels of high mobility group box 1 (HMGB1), which is associated with cell necrosis [62]. The authors also found that like HRV and human NoVs, E11 exhibits a cell type specificity of infection and infected both enterocytes and enteroendocrine cells but did not infect goblet cells [62]. It is important to note that these studies were performed on enteroids derived from premature intestine and could have a different phenotype if studies were done on enteroids derived from the intestine of older patients. 

### 5.5. Enteroid Model of Adenoviral Infection

Enteroid model systems have also been used to elucidate possible viral adaptation to the host immune system, such as in the case of adenovirus (AdV) [70,71]. Previous research has shown that while certain serotypes of adenovirus (mouse AdV-1 [70] and human AdV-C [71]) are modulated by α-defensins (antimicrobial peptides naturally secreted by Paneth cells), other serotypes (mouse AdV-2 [70] and human AdV-F) [71] are unaffected. In the study by Wilson et al., murine enteroids were used to observe an increase in enteric infection by mouse AdV-2 when exposed to α-defensins [70]. Using enteroids derived from wild-type (WT) mice and mice lacking functional α-defensins (*Mmp7^–/–^* mice), green fluorescent protein (GFP) tagged mouse AdV-2 (mouse AdV-2. IXeGFP) was microinjected into the lumen of enteroids and viral infection was measured [70]. Results indicated viral progeny was 3-fold greater in WT mice compared to *Mmp7^–/–^* mice on day 2 and 4-fold greater by day 3 post infection [70]. Additionally, quantification showed significantly higher levels of GFP-positive enteroids in WT mice compared to *Mmp7^–/–^* mice [70]. Collectively, these data suggest that in specific murine adenoviral genotypes, α-defensins can facilitate enteric infection. Further studies utilizing human intestine are required to see whether there are species-specific differences. 

## 6. Conclusions and Future Directions

While human RV and NoV remain leading causes of acute gastroenteritis, vaccine development and efficacy are still low. A major barrier in this field is the lack of understanding how these viruses are identified and interact within a host. Studies have distinguished differences in receptor recognition between NoV and RV, showing that these viruses interact with HBGAs or with specific sialic acids, respectively [69,72]. Human HBGAs are highly polymorphic and consist of ABO (blood group antigens), Lewis, and secretor ligands, which have been suggested to play a role in pathogen-host interactions. Several studies have explored various genotypes of human RV and NoV via X-ray crystallography and nuclear magnetic resonance (NMR) to determine the structure and function of HBGAs in viral infection [69,72]. Human intestinal enteroids and organoids from individuals expressing a range of HBGAs presents a unique opportunity to study potential host-pathogen interactions. 

While enteroids and organoids can be used to study host immune response, they can also give insight into the attenuation of virus replication in vitro. In one such study, Saxena et al. tested the attenuated human RV strain RV1 (used in Rotarix rotavirus vaccine) using human intestinal enteroids from secretor-negative patients [21]. After multiple attempts, RV1 viral growth consistently replicated poorly from a secretor-negative patient [21]. In utilizing these in vitro models of the intestine, the potential for a translational approach towards developing targeted vaccines for RV, NoV, or other gastroenteritis causing pathogens becomes a possibility.

With the development of novel culture systems to recapitulate the GI tract, the field of enteric viral research has witnessed many exciting new advances. Notably, these studies have yielded new in vitro models to propagate human NoV, which can be used to test and develop new antivirals and have suggested unique mechanisms by which the GI tract might sense and respond to viral infections in a virus-specific manner. Despite the progress made over the last few years using these in vitro models, the enteroid and organoid systems provide an incomplete picture of the intestinal defense mechanisms as they lack the epithelial-immune cell communication, which is known to be a critical factor in determining the outcome of GI infections. The gut-on-a-chip approach represents an exciting addition to enteroid and organoid cultures as it can mimic a higher degree of complexity by including the immune cells to the culture system, offering the possibility to investigate the different potential interactions occurring at the mucosal surface during a viral infection. Although further research is required to fully define enteric virus-GI interactions and identify host factors that could be targeted by antivirals or vaccines, the application of these human models will undoubtedly yield exciting new insights into a variety of aspects of host-virus interactions in the GI tract.
